# Inhibition of Tropomyosin Receptor Kinase A Signaling Negatively Regulates Megakaryopoiesis and induces Thrombopoiesis

**DOI:** 10.1038/s41598-019-39385-x

**Published:** 2019-02-26

**Authors:** Ayse Kizilyer, Meera V. Singh, Vir B. Singh, Sumanun Suwunnakorn, James Palis, Sanjay B. Maggirwar

**Affiliations:** 1 Department of Microbiology and Immunology, University of Rochester Medical Center, Rochester, NY United States of America; 2 Department of Pediatrics, Hematology and Oncology, University of Rochester Medical Center, Rochester, NY United States of America; 3 Present Address: Department of Molecular Biology and Genetics, Faculty of Arts and Sciences, Burdur Mehmet Akif Ersoy University, Burdur, Turkey

## Abstract

Neurotrophin signaling modulates the differentiation and function of mature blood cells. The expression of neurotrophin receptors and ligands by hematopoietic and stromal cells of the bone marrow indicates that neurotrophins have the potential to regulate hematopoietic cell fate decisions. This study investigates the role of neurotrophins and Tropomyosin receptor kinases (Trk) in the development of megakaryocytes (MKs) and their progeny cells, platelets. Results indicate that primary human MKs and MK cells lines, DAMI, Meg-01 and MO7e express TrkA, the primary receptor for Nerve Growth Factor (NGF) signaling. Activation of TrkA by NGF enhances the expansion of human MK progenitors (MKPs) and, to some extent, MKs. Whereas, inhibition of TrkA receptor by K252a leads to a 50% reduction in the number of both MKPs and MKs and is associated with a 3-fold increase in the production of platelets. In order to further confirm the role of TrkA signaling in platelet production, TrkA deficient DAMI cells were generated using CRISPR-Cas9 technology. Comparative analysis of wild-type and TrkA-deficient Dami cells revealed that loss of TrkA signaling induced apoptosis of MKs and increased platelet production. Overall, these findings support a novel role for TrkA signaling in platelet production and highlight its potential as therapeutic target for Thrombocytopenia.

## Introduction

Platelets, the smallest cellular component of circulating blood, are critically involved in hemostasis, thrombosis, and inflammation^1–4^. Diverse pathological conditions impact platelet production and/or clearance leading to aberrant platelet counts, which pose health risks due to severe hemorrhage, thrombus formation, or impaired immune response^2,5–8^. Current therapies for managing these abnormalities are neither time- nor cost-effective, and other conditions, such as infection and alloimmunization, limit their efficacy^6,9–11^. Cell-based approaches aiming at *ex vivo* platelet production are promising but necessitate further research for optimization^12,13^. In order to develop efficacious therapies, it is crucial to gain a better understanding of the molecular mechanisms underlying platelet production (thrombopoiesis).

Thrombopoiesis is a multistage process requiring megakaryocyte (MK) maturation and fragmentation in the bone marrow (BM), triggered by an array of growth factors and cytokines^14–18^. Neurotrophins are among the growth factors expressed in the bone marrow and act by binding tropomyosin receptor kinases (Trks) and/or the low affinity receptor p75NTR^19^. Of those, nerve growth factor (NGF) binds more specifically to TrkA, brain-derived neurotrophic factor (BDNF) and neurotrophin-4/5 (NT-4/5) to TrkB, and neurotrophin-3 (NT3) to TrkC^20^. Ligand binding to Trks is followed by receptor dimerization, phosphorylation of the intracellular domain via intrinsic kinase activity, and recruitment of different adaptor and effector proteins, which transmit the trophic message to downstream signaling molecules^19^. The receptor-mediated neurotrophic message is then converted to diverse cellular outcomes with the activation of PI3K (Phosphatidylinositol-3 kinase), phospholipase C gamma (PLC-γ), and MAPK pathways^19^.

Neurotrophins are essential factors for survival, proliferation, and differentiation of both neuronal and non-neuronal cells^21–24^. Previous studies have shown that neurotrophins and their receptors are expressed by both mature and immature cells of the hematopoietic system^25–29^. Although the role of neurotrophins, more specifically NGF/TrkA, in mature blood cells has been widely explored^30–41^, their functions in hematopoietic stem and progenitor cells are poorly understood. Several megakaryocytic cell lines (Meg-01, K562) are known to express TrkA^42^. When given in combination with sodium butyrate, an inducer of megakaryocytic differentiation, NGF promotes the commitment of K562 cells to the megakaryocytic lineage^43^. Treatment of erythroleukemic and megakaryocytic cell lines (HEL, Meg-J, CMK, and M07e) with a Trk receptor inhibitor, K252a, induces polyploidization and increases MK differentiation markers^44–47^. Despite the limited reports indicating a role for the neurotrophin pathway in MK development, actions of neurotrophins in subsequent platelet formation has not been elucidated. In this study, we aimed to investigate the undefined role of neurotrophin signaling in MK differentiation and platelet production. We utilized both primary cell culture and a cell line model to examine the megakaryopoietic and thrombopoietic aspects of neurotrophins, specifically NGF/TrkA signaling. Besides ligand or inhibitor-mediated modulation of TrkA, we also established TrkA-knockout DAMI cells via CRISPR-Cas9 system (clustered regularly interspaced short palindromic repeats-CRISPR associated protein 9 nuclease) to further confirm the involvement of TrkA in platelet production. Data from this study indicate that neurotrophin signaling has a bimodal role in megakaryopoiesis and thrombopoiesis. Signaling through TrkA supports megakaryopoiesis by inducing MK progenitor expansion and MK survival but subsequently suppresses MK maturation and fragmentation into platelets.

## Materials and Methods

### Reagents and antibodies

Recombinant human thrombopoietin (rhTPO), interleukin I-beta (rhIL-1β), interleukin 6 (rhIL-6), stem cell factor (rhSCF), nerve growth factor beta (rhNGF-β), and granulocyte-macrophage colony stimulating factor (rhGM-CSF) were purchased from R&D systems (Minneapolis, MN, USA). K252a was purchased from Calbiochem (San Diego, CA, USA). The following fluorochrome-conjugated anti-human antibodies were used for flow cytometry analysis: FITC-labelled human lineage cocktail 4 (CD2, CD3, CD4, CD7, CD8, CD10, CD11b, CD14, CD19, CD20, CD56, CD235a), Sca-1-FITC, CD34-PE Cy7, CD41-APC, TrkA-PE (all from BD Pharmingen, San Diego, CA, USA), CD61-AF 647 were obtained from Biolegend (San Diego, CA, USA). The nuclear dyes, 7-Aminoactinomycin D (7-AAD) and propidium iodine (PI), were also from BD Pharmingen.

### Cell lines and culture

Unless otherwise stated, all cell lines used in this study were cultured in media containing 10% fetal bovine serum (FBS), 2 mM glutamine, and 1% penicillin-streptomycin-gentamicin (PSG), and maintained at 37 °C in a 5% CO_2_ humidified atmosphere. Specifically, Meg-01, M07e, and Dami cells were obtained from Dr. Richard Phipps (University of Rochester Medical Center, Rochester, NY, USA) and cultured in RPMI 1640 (Roswell Park Memorial Institute Medium). 10 ng/ml rhGM-CSF was used to induce proliferation of M07e cells. Human embryonic kidney (HEK 293 T) cells were obtained from Invitrogen/Life Technologies (Grand Island, NY, USA), and cultured in Dulbecco’s Modified Eagle Medium (DMEM).

### *Ex vivo* differentiation of primary human megakaryocytes

CD34+ cells derived from BM or umbilical cord blood were purchased from Allcells (Emeryville, CA, USA), and differentiated as previously described^48^. For inducing MK progenitor (MKP) cell differentiation and expansion, CD34+ cells were cultured for 7 days in Iscove’s modified Dulbecco media (IMDM, Invitrogen/Life Technologies) containing 20% BIT 9500 (Stem Cell Technologies, Tukwila, WA, USA), 0.8% low density lipoprotein (LDL, L-8292, Sigma-Aldrich Corp., St. Louis, MO, USA), 100 ng/mL rhTPO, 10 ng/mL rhIL-1β, 10 ng/mL rhIL-6, and 50 ng/mL rhSCF. At day 7, cells were plated in fresh media without rhSCF, and either used for experiments or further cultured for 7 days to differentiate into MKs.

### Immunoblotting assay

Protein lysates were prepared as previously described^49^. Protein content was quantified by Bradford assay, and samples were separated in 7.5% sodium dodecyl sulfate-polyacrylamide gel electrophoresis (SDS-PAGE). Proteins were electrophoretically transferred onto Hybond ECL nitrocellulose membrane (GE Healthcare Bio-Sciences Corporation, Piscataway, NJ, USA), and incubated with primary antibodies raised against human TrkA (sc-118, 1:1000), TrkB (sc-20542, sc-12, 1:1000), TrkC (sc-14025, sc-117, 1:1000), p75NTR (sc-8317, 1:1000), phospho-Trk (Tyr680/Tyr681, sc-7996-R, 1:1000), Erk1 (sc-94, 1:1000), phospho-Erk (sc-7383, 1:1000), α-tubulin (sc-8035, 1:1000; all from Santa Cruz Biotechnologies Inc., Santa Cruz, CA, USA), Akt (9272, 1:2000), and phospho-Akt (4060, 1:2000, all from Cell Signaling Technology, Danvers, MA, USA). Species-specific infrared secondary antibodies (Li-Cor Biosciences, Lincoln, NE) were used to detect bound antibodies, followed by visualization using Li-Cor Odyssey Infrared Imaging System. Densitometry was performed using Image Studio Lite software (Li-Cor Biosciences).

### Reverse transcriptase polymerase chain reaction (RT-PCR)

Total RNA was extracted from cells using Trizol reagent according to the manufacturer’s instructions (Ambion/Life Technologies, Carlsbad, CA, USA). 1 µg RNA was DNAse I-treated (Invitrogen/Life Technologies, Carlsbad, CA, USA) and subjected to cDNA synthesis by iScript cDNA synthesis kit (Biorad, Hercules, California, USA). Primer pairs listed in Table 1 were used to amplify the cDNA of human TrkA, TrkB, TrkC, p75NTR, NGF, and GAPDH. Trk, tropomyosin receptor kinase; p75NTR, p75 neurotrophin receptor; GAPDH, glyceraldehyde 3-phosphate dehydrogenase; sgTrkA, single guide RNA for TrkA; ssODN, single-stranded oligodeoxynucleotide.

**Table 1:** Primer list for non-quantitative reverse transcriptase PCR analysis.

Primer	5′-3′ sequence
TrkA forward	CTGGGCAGAGAACGATGTGG
TrkA reverse	CATTGAAGAGCCAGCGCAGA
TrkB forward	AATGACATCGGGGACACCAC
TrkB reverse	ATCCCACCACAGACGCAATC
TrkC forward	CAACTGCAGCTGTGACATCC
TrkC reverse	AACAGCGTTGTCACCCTCTC
p75NTR forward	CCAGTCGTCTCATCCTGGTAG
p75NTR reverse	CCAGTCGTCTCATCCTGGTAG
GAPDH forward	CCTGCACCACCAACTGCTTA
GAPDH reverse	CCATCACGCCACAGTTTCC
sgTrkA forward	CACCGGACTGCGATGCACCCGGGAT
sgTrkA reverse	AAACATCCCGGGTGCATCGCAGTCC
TrkA ssODN 1	TGCCCCGATGCCTGCTGCCCCCACGGCTCCTCGGGACTGCGATGCACCCGGATGGGGCCCTGGATAGCCTCCACCACCTGCCCGGCGCA
TrkA ssODN 2	TGCGCCGGGCAGGTGGTGGAGGCTATCCAGGGCCCCATCCGGGTGCATCGCAGTCCCGAGGAGCCGTGGGGGCAGCAGGCATCGGGGCA
TrkA-RFLP forward	CTGCTGGCTTGGCTGATACT
TrkA-RFLP reverse	ACTGCAATGCCTGCCTGT

### Flow cytometric analysis of megakaryocytes, megakaryocyte progenitors, and culture-derived platelets

Dami cells were plated at 1 × 10^5^ cells in 1 ml media per well in a 24-well plate and incubated for 72 h under the specified treatment conditions (200 ng/ml rhNGF-β; 200 nM K252a). At the indicated time points, cells were homogenously mixed and 50 µl of cell suspension was stained first with anti-human CD61-AF 647 antibody for 20 min followed by staining with 7-AAD for 10 min at room temperature (RT). Absolute counts of MKs and platelet-like particles (PLPs) in 10 µl volume were quantified using a Accuri C6 flow cytometer (BD Biosciences, CA, USA) as previously described^48^. Data were analyzed using FlowJo software (TreeStar, Ashland, OR, USA). CD61+ 7-AAD− Dami cells are designated as intact MKs. PLPs are distinguished as CD61+ 7-AAD− cells falling into the gate based on forward and side scatter of human whole blood platelets^50^. To normalize well-to-well variation, PLP counts were calculated as PLP per MK.

For detection of *ex vivo* differentiated MKPs and MKs, cells were seeded at 1 × 10^5^ cells/ml media (IMDM, 10% BIT 9500, rhIL-6, rhIL-1β, 100 ng/ml rhTPO, 0.8% LDL) into a 24-well plate. At the indicated time points, cells were homogenously mixed, 350 µl of cell suspension was transferred from each well into two separate tubes, and stained for culture-derived MKPs/MKs and platelets. After staining, cells were washed with PBS and resuspended in 50 µl volume. Using Accuri C6, absolute cell count in 10 µl was determined. Following compensation, unstained cells and fluorescence minus one controls were used to properly gate each cell type. Using FITC-labelled human lineage cocktail and anti-human Sca-1 antibodies, Lin- Sca-1− cells were selected. Among those, CD34+ CD41+ cells were gated as MKPs and CD34−CD41+ cells were identified as MKs. Culture-derived platelets were detected by CD61+ PI- staining in the size gate of human platelets and platelet per MK counts were used for evaluation.

### Cell cycle analysis

Cell cycle analysis was performed using a FITC-BrdU Flow Kit (BD Biosciences, CA, USA). Briefly, serum starvation (1 h) was performed for synchronization of cells at G0/G1 phase. Cells were subsequently split at 2.05 × 10^5^ in 0.5 ml media (RPMI with 10% FBS and 1% PSG) in a 48-well plate and cultured 24 h. Cells were pulsed with 5 µl BrdU (1 mM) for 30 min prior to the end of 24 h incubation, then processed according to the manufacturer’s protocol. After flow cytometric acquisition, cell fractions at S, G0/G1, and G2/M phases were determined based on the intensities of anti-BrdU-FITC and total nuclear dye, 7-AAD.

### Apoptosis assay

Annexin V-PE 7-AAD Apoptosis Kit from BD Biosciences was used to determine Annexin V+ early and late apoptotic cells. Briefly, 1 × 10^5^ cells were immunostained with both Annexin V-PE and cell impermeable nuclear dye, 7-AAD following the manufacturer’s instructions. Sample analysis was performed in Accuri C6 flow cytometer by acquiring 10,000 events/sample. Annexin V+  7-AAD− cells were considered early apoptotic cells while double positive cells were considered late apoptotic cells. Annexin V− 7-AAD + cells were considered dead.

### Generation of TrkA deficient cells by CRISPR

These were generated by employing CRISPR technology^51^. Briefly, oligos for TrkA guide RNA (sgTrkA forward and reverse, Table 1) were designed to contain 20 nucleotides (NG_007493: 50324-50344 nucleotides) with a 5′-GGG protospacer adjacent motif (PAM) located in exon 1 of TrkA and 5′ flanking sequence complementary to a BbsI precut vector. Oligos were purchased from Integrated DNA Technologies (Coralville, IA, USA), annealed, and cloned into the pSpCas9 (BB)-2A-GFP plasmid (Addgene, #48138) as described by Ran *et al*.^51^. This system allows for the coexpression of sgTrkA and Cas9 from a single construct and use of Enhanced Green Fluorescent Protein (EGFP) as a reporter. Dami cells were nucleofected with the CRISPR construct along with the repair template (TrkA ssODN Primer 1 and 2 in Table 1) using Nucleofection Kit C and the X-05 program settings in Amaxa Nucleofector I device (Lonza/Amaxa Biosystems, Cologne, Germany), according to the manufacturer’s instructions. Nucleofected cells were then seeded into a 12-well plate containing 1.5 ml complete media and cultured for 24 h prior to sorting.

For fluorescence-activated cell sorting (FACS), GFP-positive Dami cells were sorted using a 130 µm nozzle at BD FACSAria™ Cell Sorting System. Sorted cells were cultured for 24 h and seeded in a 96-well plate by serial limiting dilution. Single cell colonies were grown, expanded, and pre-screened for the presence of expected nucleotide change by Restriction Fragment Length Polymorphism Analysis of PCR-Amplified Fragments (PCR-RFLP). For this purpose, the region flanking the mutation site in TrkA DNA sequence was PCR-amplified using TrkA-RFLP forward and reverse primers (Table 1). Resultant 201 nucleotide base pair PCR product was subjected to XmaI digestion. Uncleaved band following XmaI digestion indicated successful deletion of the guanine nucleotide. Clones preselected by PCR-RFLP were further sequenced for validation.

### Statistical analysis

Statistical analysis was performed using Graphpad Prism Software (La Jolla, CA, USA). Student’s t test was used to compare means of two groups. Data from multiple groups were analyzed by one-way analysis of variance (ANOVA) or two-way ANOVA, where appropriate, and followed by Bonferroni test for multiple comparisons. Results were represented as mean ± standard error of means (SEM). P values equal or lower than 0.05 were considered statistically significant.

## Results

### Neurotrophin receptors are expressed both in primary human megakaryocytes and megakaryocytic cell lines

**Figure 1:**
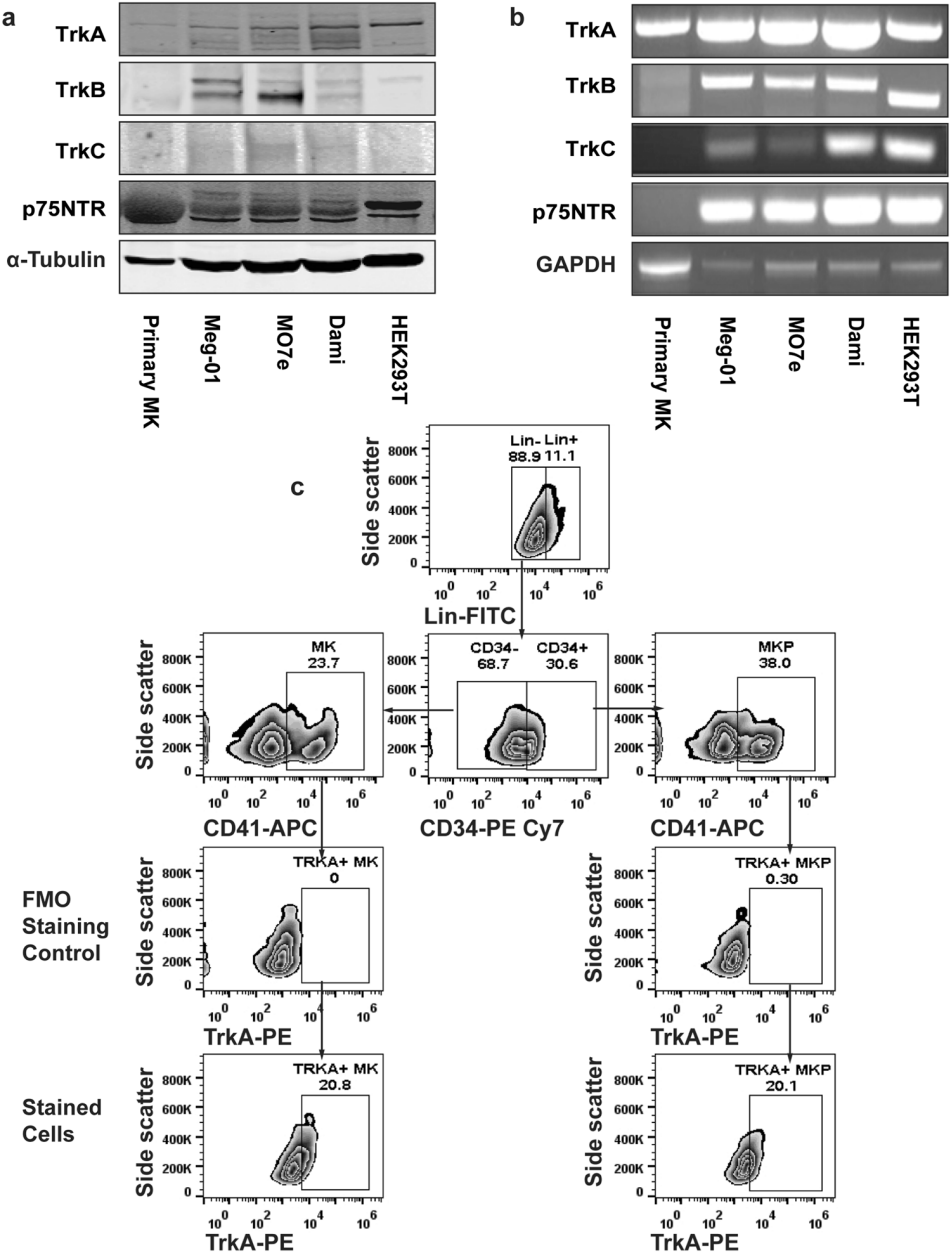
Neurotrophin receptors are expressed both in human primary MKPs, MKs and MK cell lines. (**a**) Human umbilical cord blood-derived CD34+ cells were differentiated in a suspension culture containing IMDM, 20% BIT 9500, 8% Low Density Lipoprotein, 100 ng/mL rhTPO, 10 ng/mL rhIL-1β, 10 ng/mL rhIL-6, for 7 days with 50 ng/mL rhSCF, and another 7 days without rhSCF. At day 14, the final MK-rich (73%) cell suspension was used to prepare total RNA and protein lysates. Protein lysates were also made from Dami, meg-01 and MO7e cell lines. HEK293T cells were used as a positive control due to endogenous expression of all neurotrophin receptor types. At least 30 µg protein from each cell type was resolved via SDS-PAGE and subjected to immunoblotting assays for the indicated proteins. The blot shown is representative of three experiments. (**b**) Non-quantitative, reverse transcriptase PCR was used to detect the expression of neurotrophin receptors in *ex vivo* differentiated human MKs and MK cell lines. Glyceraldehyde 3-phosphate dehydrogenase (GAPDH) mRNA was amplified to normalize the neurotrophin receptor expression level. The results shown are representative of three independent experiments. (**c**) Human bone-marrow derived CD34+ cells were differentiated *ex vivo* for 7 days in the presence of IMDM, 20% BIT 9500, 8% Low Density Lipoprotein, 100 ng/mL rhTPO, 10 ng/mL IL-1β, 10 ng/mL rhIL-6, and 50 ng/mL rhSCF. The Day 7 culture containing 38% MKPs (Lin-CD34+CD41+) and 23.7% MKs (Lin-CD34−CD41+) was analyzed for TrkA surface expression by flow cytometry. Graphs of fluorescence minus one control for TrkA staining are shown as staining controls for TrkA antibody. The data shows the presence of TrkA expression in both MKPs (20.1%) and MKs (20.8%). All treatments were done in triplicates in each experiment and data are representative of two independent experiments.

First, we characterized the expression of neurotrophin receptors in primary human MKs and several MK cell lines. Primary human MKs were obtained by *ex vivo* differentiation of umbilical cord blood-derived CD34+ cells with a two-stage culture method. Human megakaryocytic cell lines Meg-01, M07e, and Dami, represent different stages of MK differentiation. Although TrkA expression of human CD34+ cells and Meg-01 cells has been previously reported^25,28,42^, we performed a comprehensive expression analysis for the neurotrophin receptors TrkA, TrkB, TrkC, and p75NTR in primary MKs and MK cell lines. HEK293T cells were used as a positive control since a previous report from our group and the current study revealed endogenous expression of all neurotrophin receptor subtypes in HEK293Ts^52^. Immunoblot (Fig. 1a) and non-quantitative RT-PCR (Fig. 1b) analysis demonstrated that TrkA is the common isoform expressed by both MK cell lines and primary MKs, whereas TrkC was only expressed in the MK cell lines. Primary MKs showed a protein smear for p75NTR, however we were unable to detect any RNA for it by RT PCR. Furthermore, TrkB expression was not detected in primary MKs (Fig. 1a). Since TrkA is the only neurotrophin receptor subtype expressed in both normal human MKs and human MK cell lines, we focused on the NGF-TrkA signaling axis for the remainder of this study.

To further explore the distribution of TrkA in immature MKPs and MKs, human BM-derived CD34+ cells were differentiated in MKP expansion media. After a 7-day culture, cells were subsequently analyzed for TrkA expression via flow cytometry, where MKP (38%) and MK (23.7%) cells were initially delineated by CD34 and CD41 expression (Fig. 1c). We ultimately observed that 20.8% of MKs and 20.1% of MKPs have surface expression of TrkA.

### NGF/TrkA signaling supports MKP expansion but blocks platelet production

**Figure 2:**
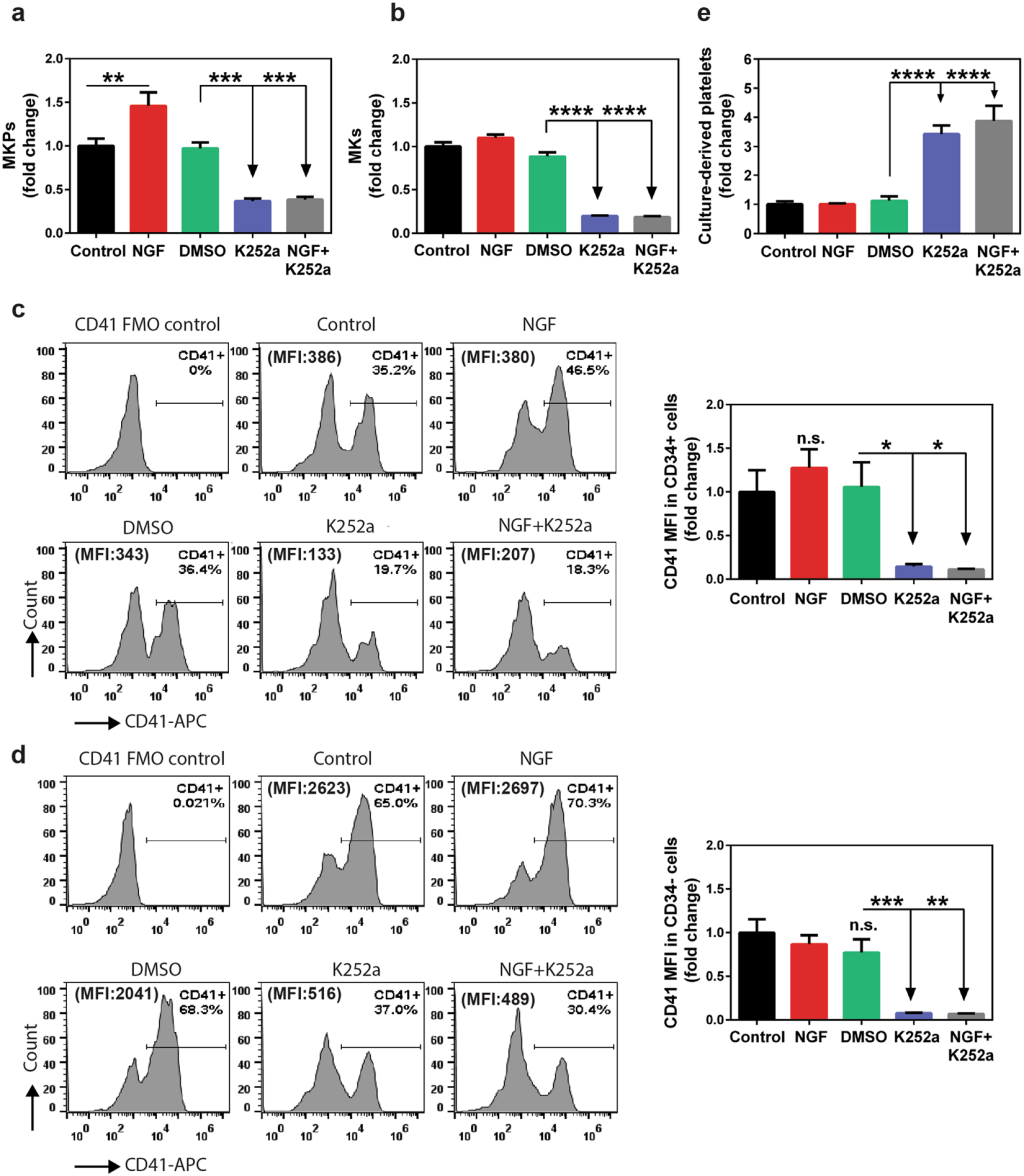
NGF/TrkA signaling supports the expansion of MKPs but blocks platelet production. Human MKPs and MKs differentiated from BM-derived CD34+ cells were plated in a medium containing 100 ng/mL rhTPO, 10 ng/mL rhIL-1β, 10 ng/mL rhIL-6 and treated with both NGF (200 ng/ml) and K252a (200 nM), alone and in combination. The control cultures were treated with phosphate-buffered saline (PBS). Following 72 h incubation, 350 µl cell suspension was stained with antibodies against MK lineage-specific markers, and each sample was analyzed for MKP, MK, and platelet-like particle counts by flow cytometry. The addition of NGF to the cytokine cocktail led to further expansion of MKPs (**a**) and a slight increase in MK (**b**) counts, but did not induce a significant change in CD41 surface expression among neither CD34+ (**c**) nor CD34- (**d**) cells comprising MKPs and MKs, respectively In contrast, K252a treatment caused a decline in both MKP (**a**) and MK (**b**) counts while decreasing their CD41 expression (**c**,**d**). Panels (c and d) also show histogram plot from one representative experiment. (**f**) NGF did not alter platelet per MK yield whereas K252a augmented platelet production. All treatments were done in triplicates in each experiment and data are representative of two independent experiments. Results presented are the mean ± SEM and analyzed by one-way ANOVA, **p < 0.01, ***p < 0.001, ****p < 0.0001. n.s. denotes statistically not significant.

Since TrkA was found to be expressed on MKPs and MKs, we next examined whether NGF-induced TrkA signaling has a functional role in expansion and differentiation of MK lineage cells. For this purpose, a mixture of culture-derived MKPs and MKs was generated from human BM-derived CD34+ cells and seeded in a multi-well plate containing serum-free medium supplemented with TPO, IL-6, IL-1β, and hLDL. Cells were cultured for 72 h in the presence of NGF or K252a. Absolute counts of MKPs and MKs were assessed by flow cytometry. As demonstrated in Fig. 2a, NGF treatment significantly enhanced the expansion of MKPs (p < 0.01) compared to control, however had no effect on MK counts (Fig. 2b). In contrast, treatment with K252a, alone or in combination with NGF, significantly reduced the number of both MKPs (p < 0.001, Fig. 2a) and MKs (p < 0.0001, Fig. 2b).

The increase in MKP numbers following NGF stimulation might be a result of commitment of early progenitors to MK lineage, or increased clonal expansion of lineage committed cells. In order to assess this possibility, we analyzed the surface expression of CD41 among CD34+ cell population (Fig. 2c), which is a lineage-specific marker upregulated during MK differentiation. While, NGF treatment alone did not significantly increase the median fluorescence intensity (MFI) of CD41 in MKPs (Fig. 2c), MKPs generated in presence of K252a alone (p < 0.05) or NGF and K252a combination (p < 0.05) showed significantly decreased levels of CD41 expression compared to control or DMSO In addition, CD41 MFI among CD34− cells (Fig. 2d) encompassing MKs was not altered by NGF however it was significantly reduced by K252a treatment (K252a vs DMSO, p < 0.01; NGF + K252a vs DMSO, p < 0.001). Altogether, these results suggest that inhibition of TrkA signaling by K252a supports clonal expansion of MKPs but does not promote MK lineage commitment. K252a-mediated reduction in CD41 MFI among CD34+ and CD34− cells indicates that TrkA-mediated signaling is important for preservation of MK lineage markers.

Lastly, we examined whether TrkA signaling impacts platelet production from *ex vivo* differentiated MKs. We therefore quantified the culture-derived platelet content in the aforementioned treatment conditions. We did not observe any change in the number of platelets with NGF at 72 h (Fig. 2e). However, K252a either alone (p < 0.0001), or in combination with NGF (p < 0.0001), resulted in a 3-fold increase in platelet generation. It should be noted that the indolocarbazole K252a inhibits tyrosine kinase activity or TrkA regardless of its binding to NGF^53^. Collectively, these results strongly suggest that inhibition of TrkA signaling promotes thrombopoiesis.

### Dami cells serve as a model to study TrkA signaling

As our work with primary MK cultures indicated that TrkA signaling differentially modulates megakaryopoiesis and subsequent thrombopoiesis (Fig. 2), we were prompted to further investigate the regulatory role of TrkA-mediated signaling in these two processes. We performed subsequent mechanistic studies in Dami cells, which express TrkA (Fig. 1) and serve as a useful *in vitro* model to study MK differentiation and platelet production^54,55^.

**Figure 3:**
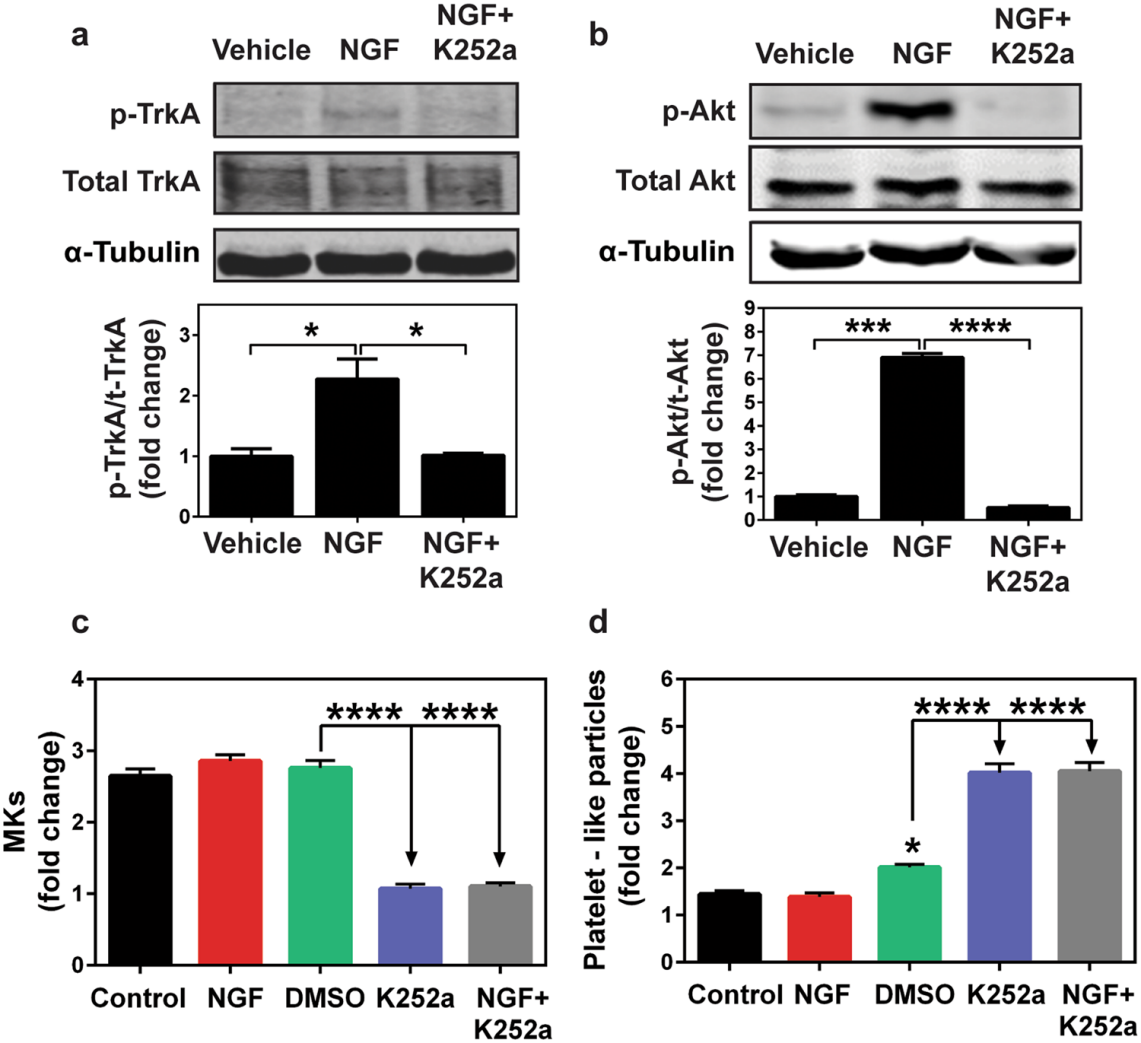
Dami cells serve as a model to study TrkA signaling. Dami cells were subjected to 1 h serum starvation and pre-treated with 200 nM of K252a, a Trk inhibitor, for 15 min prior to the end of 1 h incubation. Then cells were incubated with 200 ng/ml rhNGF for 5 min. Total cell extracts were obtained and analyzed by immunoblotting using the indicated antibodies. Representative immunoblotting images show the NGF-induced phosphorylation of (**a**) TrkA (Tyr680/Tyr681, 140 kD) and simultaneous phosphorylation of downstream (**b**) Akt (Ser473, 60 kD). 15 min pre-treatment of Dami cells with 200 nM K252a, a Trk inhibitor, blocks the phosphorylation of both (**a**) TrkA and downstream (**b**) Akt proteins. Densitometric analysis for TrkA and Akt levels were expressed as the ratio of phosphorylated form to total protein normalized by α-tubulin. The bar graphs show the data as the mean fold change ± SEM of immunoblot images from three independent experiments. Data were analyzed by one-way ANOVA (**a**,**b**). *p < 0.05, ***p < 0.001, ****p < 0.0001. (**c)** Dami cells were seeded at a density of 1 × 10^5^ cells/ml in RPMI 1640, containing 5% FBS, 1% PSG, and then treated with 200 ng/ml rhNGF and 200 nM K252a, both alone or in combination. DMSO served as a vehicle control for K252a. The treatments were terminated at 48 h and 50 µl cell suspension was stained with anti-human CD61-AF647 antibody and nuclear dye, 7-AAD. Counts of intact Dami cells (7-AAD−CD61+) and platelet-like particles (7-AAD−CD61+) in 10 µl volume were determined by flow cytometry. Platelet-like particle counts were normalized by the number of MKs measured in each sample volume. Measures of total MK counts revealed a slight increase with NGF (statistically not significant.) and a significant decrease in the presence of K252a. (**d**) Inversely correlated with decreased MK counts in K252a-containing culture, platelet-like particle counts showed a statistically significant increase following exposure to K252a for 48 h. Data in (**c**,**d**) were analyzed by one-way ANOVA; (*p < 0.05, ****p < 0.0001).

We first assessed the functionality of TrkA receptors in Dami cells by performing short-term NGF exposure. Dami cells were subjected to serum withdrawal for 1 h, then exposed to NGF (200 ng/ml) for 5 min in serum free conditions. Whole cell lysates were generated and subsequently probed with total and phosphoprotein-specific antibodies against TrkA and Akt. 5 min NGF exposure induced the tyrosine phosphorylation of TrkA at Tyr680/Tyr681 (Tyr674/Tyr675 on human TrkA isoform) (Fig. 3a). Moreover, phosphorylation of TrkA was associated with the activation of PI3K/Akt, which is a common downstream mediator of TrkA signaling (Fig. 3b)^19^. As expected, Akt phosphorylation was abrogated by 1 h pretreatment with LY294002 (50 µM), a PI3K inhibitor (Supplementary Fig. 2d). In contrast, 15 min pretreatment with K252a (200 nM) blocked the phosphorylation of TrkA (Fig. 3a) and Akt (Fig. 3b) both, suggesting that the inhibition of TrkA activity by K252a also results in attenuation of downstream signaling events.

Next, we investigated the effect of NGF or K252a treatment on Dami cells in order to validate that Dami cells recapitulate the response observed in primary human MKs (Fig. 2b). Dami cells were seeded in a multi-well plate containing RPMI with 5% FBS and cultured for 48 h with the respective treatments. Flow cytometric analysis showed that similar to our observations in primary MK culture, NGF did not affect Dami cell counts, whereas K252a caused approximately 3-fold decrease in cell count (p < 0.0001) at 48 h (Fig. 3c). In addition, K252a induced a significant increase (~2–4 fold) in platelet-like particle counts compared to DMSO control at 48 h (p < 0.0001, Fig. 3d). Based on these findings, we concluded that Dami cells have functional TrkA receptors which can be modulated through inhibitions of TrkA phosphorylation by K252a and that these cells recapitulate the response we observed in primary MKs after activation or inhibition of TrkA signaling.

### Inhibition of TrkA signaling by K252a promotes platelet production by inducing cell cycle arrest and apoptosis in Dami cells

**Figure 4:**
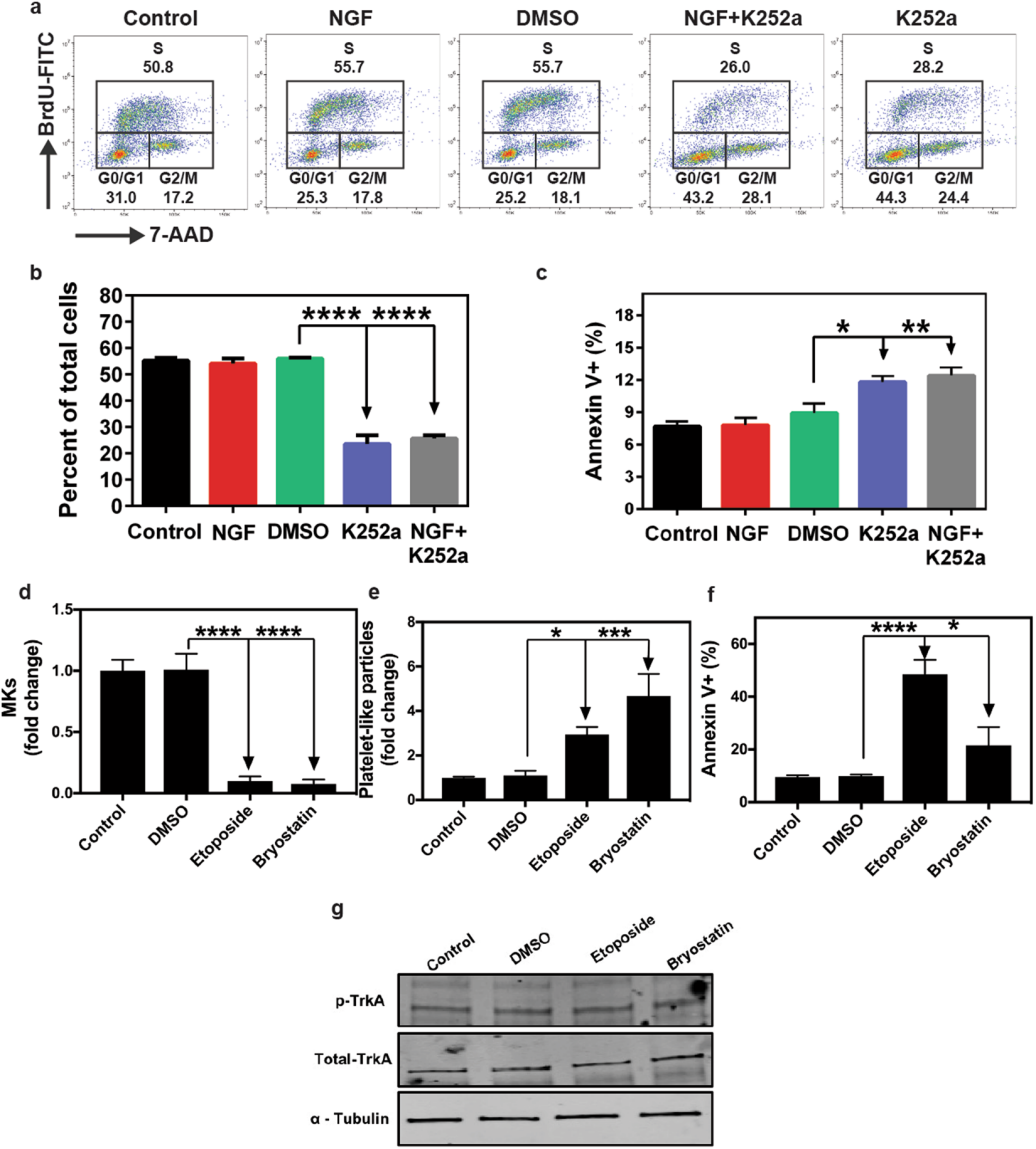
Inhibition of TrkA signaling by K252a causes cell cycle arrest and increased apoptosis in Dami cells. (**a**,**b**) Cell cycle analysis was performed by seeding 2.5 × 10^5^ cells in a 48-well plate after 1 h serum starvation and pulsing them with BrdU for 30 min prior to the end of 24 h incubation. After staining with anti-BrdU-FITC and total DNA dye, 7-AAD, cells were analyzed by flow cytometry. BrdU-FITC+ 7-AAD+ cells represent the actively proliferating cells. NGF does not cause a significant change in cell proliferation, however, K252a treatment alone or in combination with NGF leads to cell cycle arrest at G0/G1 and G2/M phases and generates a 50% reduction in cell proliferation. (**c**) Slow growth rate with K252a was also due to increased apoptosis-mediated cell death. Results presented are the mean ± SEM of at least three independent experiments. Data in (**b**,**c**) were analyzed by one-way ANOVA (*p < 0.05, **p < 0.01, ****p < 0.0001). (**d**) Fold change in MK counts (**e**) Fold change in PLP count (**f**) percetage of Annexin V+ cells and (**g**) TrkA phosphorylation, upon treatment with Etoposide and Bryostatin.

During thrombopoiesis, MKs undergo endomitosis, followed by proplatelet/platelet production and subsequent apoptosis. Since, inhibition of TrkA signaling by K252a leads to increased PLP production, we sought to analyze cell proliferation and survival in Dami cells under those treatment conditions. Through flow cytometric analysis of BrdU incorporation and total DNA staining, we determined that K252a treatment arrests the cell cycle at G0/G1 and G2/M phases (Fig. 4a), which leads to a 50% reduction in the percentage of actively proliferating cells (p < 0.0001, Fig. 4b). Furthermore, K252a treatment elevated the frequency of total Annexin V+ cells, which is indicative of early and late apoptotic cells (p < 0.05 for DMSO vs K252a, Fig. 4c). Collectively, these data suggest that TrkA inhibition by K252A indeed drives MK cells towards decreased cell proliferation, cell survival, leading to higher levels of platelet production.

It is well known that platelet production is associated with apoptosis^56^. In order to investigate if, other known inducers of apoptosis, also affect TrkA phosphorylation and cause a similar effect on MK counts as well as PLP production, Dami cells were treated with two drugs, Etoposide (1 μM) and Bryostatin (10 nM) for 72 hours as described before^57,58^. Results showed, that Etoposide and Bryostatin both significantly reduce MK counts and increase PLP production (to levels similar to those obtained by K252a treatment, Fig. 4d,e, p < 0.0001 as compared DMSO), without any effect on TrkA phosphorylation (Fig. 4g). However, it is noteworthy that these drugs caused significantly more apoptosis than K252a (Fig. 4f), and that there is no correlation between percentage of apoptosis induced by these drugs (Annexin V+ cells) and PLP counts (Supplementary Fig. 4d). These results support a previously well-known notion that increased apoptosis can induce an increase in PLP production. However, the same cannot be said about K252a mediated increase in PLP production, because, K252a causes similar increase in PLP production, via modulating TrkA phsphorylation and at a much less extent of cellular apoptosis.

### Genetic knock-out of TrkA expression by CRISPR induces decrease in cell proliferation and survival of Dami cells and increases platelet production, similar to K252a treatment

**Figure 5:**
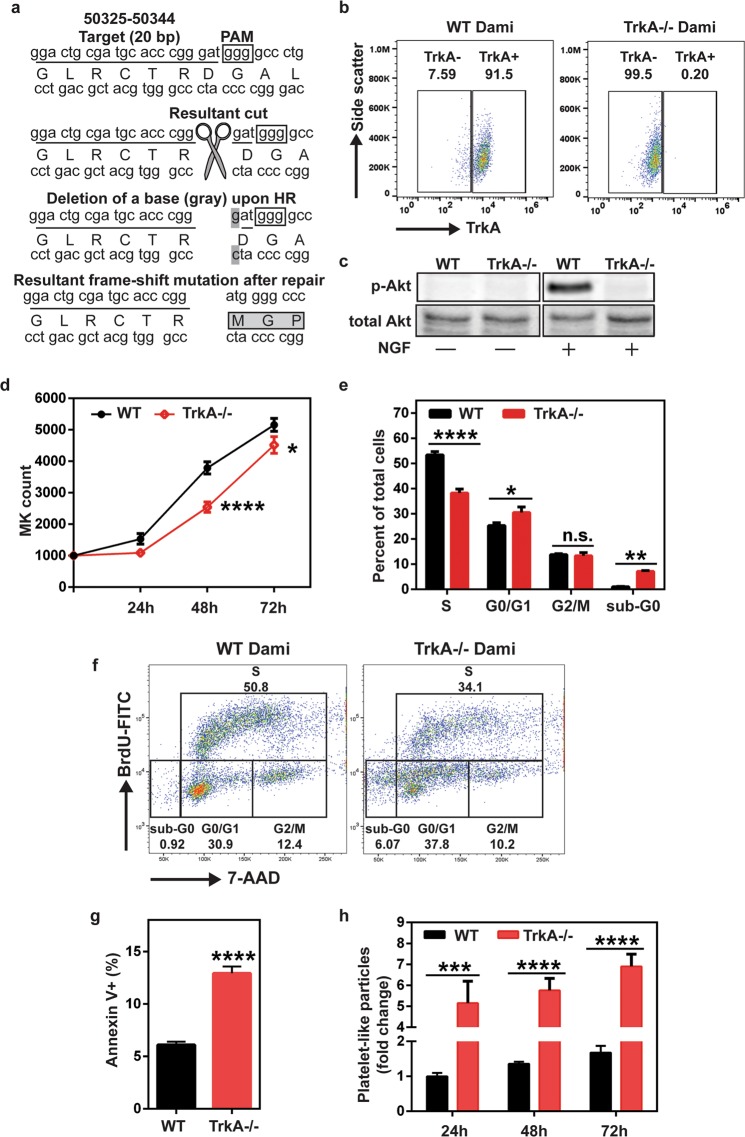
TrkA deficient Dami cells exhibit decreased growth rate and cell survival as well as increased platelet production. (**a**) For CRISPR/Cas9-mediated targeting of TrkA DNA, TrkA guide RNA (sgTrkA) was designed as complementary to a 20 nucleotide sequence (underlined; NG_007493: 50324-50344) with a 5′- GGG protospacer adjacent motif (PAM) (in square) located in the first exon of TrkA DNA. sgTrkA sequence was then inserted into an SpCas9(BB)-2A-GFP expression plasmid and nucleofected into Dami cells along with a 100 bp repair template (listed in Methods). In cells transfected with the expression plasmid and the repair template, Cas9 will be directed to the target sequence by sgTrkA due complementary base pairing and recognizes the 5′-GGG PAM sequence. Cas9 then introduces a double strand cut three nucleotides upstream of the GGG motif, which will trigger the DNA repair response. Repair by utilization of the template sequence leads to a single nucleotide (gray) deletion in the region which results in a frame-shift (DGA > MGP) in the TrkA mRNA and impairs the expression of TrkA protein. (**b**) Loss of TrkA expression was validated through flow cytometry by immunostaining wild-type (WT) and TrkA knockout (TrkA−/−) cells for TrkA surface expression. (**c**) Loss of NGF-mediated phosphorylation of Akt also confirmed the absence of functional TrkA signaling in TrkA−/− cells. (**d**) TrkA knockout cells showed a decreased growth rate compared to their WT counterparts. (**e**,**f**) Cell cycle analysis demonstrated a reduction in S phase cells coupled with an increase in G0/G1 and sub-G0 cell percentage. (**g**) Loss of TrkA expression also led to increased apoptotic death after a 24 h incubation. (**h**) TrkA−/− cells exhibited increased baseline production of platelet like particles. Results were plotted as mean with SEM from three independent experiments. Data in (**d**,**e** and **h**) were analyzed by two-way ANOVA; Student’s t test was used to evaluate the data in (**g**). (n.s. (not significant), *p < 0.05, **p < 0.01, ****p < 0.0001).

K252a is a robust inhibitor of tyrosine kinases (including PKC, PKA, PKG, MLCK, CaMK, Trks), however it is highly selective for Trk receptor subtypes in a concentration range of 10–200 nM^59–62^. Since the highest concentration of this range (i.e. 200 nM) was used in our experiments, we created a TrkA knockout Dami cell line (TrkA−/−) using CRISPR/Cas9 gene knockout system (Fig. 5a) to validate that K252a-mediated changes in MK cell proliferation, and platelet production were due to the inhibition of TrkA signaling^51^. While wild-type Dami cells have homogenous surface expression of TrkA (91.5%, Fig. 5b), our knockout strategy caused complete loss of TrkA as shown by flow cytometry analysis of TrkA surface expression (Fig. 5b) and loss of TrkA-mediated Akt phosphorylation (Fig. 5c).

After establishing TrkA−/− cells, we performed a functional analysis to assess the growth, survival and baseline platelet production of these cells compared to WT Dami cells. Equal number of WT and TrkA−/− Dami cells were plated in RPMI medium containing 5% FBS and cultured for 72 h. As shown in Fig. 5d, TrkA−/− cell culture displayed a slower growth rate relative to WT Dami cells (p < 0.001 at 48 h) as seen with K252a treated WT Dami cells (Fig. 4a,b). Cell cycle analysis (Fig. 5e,f) revealed that the loss of TrkA led to the increased percentage of sub-G0 (p < 0.01, dead cells) and G0/G1(p < 0.05) cells while S phase cells (36.7% vs 53% in TrkA−/− and WT, respectively, p < 0.0001) was significantly decreased We also assessed the percentage of apoptotic cells and observed a 2-fold higher Annexin V+ cell in TrkA−/− cells compared to WT cell culture (p < 0.0001, Fig. 5g). These findings suggest that TrkA is important for cell cycle initiation and survival for MKs. These data also corroborate with that shown in Fig. 3c, indicating that NGF-induced TrkA signaling is necessary but not sufficient to increase Dami cell proliferation.

Loss of TrkA-mediated signaling also impacts platelet production. TrkA−/− cells exhibited a higher baseline level of PLP production, (approximately 5–7 fold increase) relative to WT Dami cells at the selected time points (p < 0.0001, Fig. 5h). Based on the decreased cell growth, survival, and increased platelet production observed in TrkA−/− cells, we concluded that loss of TrkA generates a phenotype similar to the one in K252a-treated WT Dami cells (Figs 3 and 4). Overall, these data indicate that TrkA signaling is necessary for MK cell proliferation and survival, while blocking platelet production.

### TrkA signaling is necessary for platelet production as well as MK proliferation

**Figure 6:**
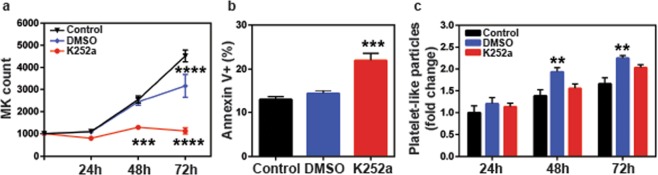
K252a modulates thrombopoiesis through TrkA inhibition. TrkA−/− Dami cells were treated with 200 nM K252a, or vehicle (DMSO) for 72 h. Cell suspension from each condition was analyzed by flow cytometry at indicated time points for MK content and intact platelet-like particles. Additionally, following 24 h incubation, 1 × 10^5^ cells were removed from each well and stained with Annexin V-PE and 7-AAD for apoptotic cell analysis. The data show that K252a is still able to cause a decrease in (**a**) cell growth rate and increase in (**b**) apoptotic cell percentage in TrkA knockout cells, implying that K252a does not only target TrkA but affects additional intrinsic factors. However, (**c**) K252a is no longer to increase the platelet production in TrkA knockout cells. Results were plotted as mean with SEM of at least three independent experiments. Data in (**a**,**c**) were analyzed by two-way ANOVA; one-way ANOVA was used to evaluate the data in (**b**). (**p < 0.01, ***p < 0.001, ****p < 0.0001).

In order to further confirm that the effects of K252a are indeed through inhibition of TrkA signaling, the TrkA−/− cells were treated with K252a for up to 72 hours and cell counts, cell survival as well as PLP counts were measured. While, DMSO (vehicle) treatment decreased cell growth (p < 0.0001, Fig. 6a), this effect was not due to increased apoptosis (Fig. 6b). Beyond DMSO’s effects, K252a significantly decreased cell growth (DMSO vs K252a, p < 0.001 at 48 h, p < 0.0001 at 72 h, Fig. 6a) and this was associated with significant increase in Annexin V+ apoptotic cells (p < 0.0001, Fig. 6b). These results as well as the data shown in Fig. 5, clearly indicate that TrkA signaling is essential for MK cell proliferation/survival and the absence of this receptor renders the TrkA−/− cells, especially susceptible to toxic effects of DMSO as well as K252a (even more so than WT Dami cells). Interestingly, while DMSO led to a significant increase in platelet-like particle counts (p < 0.01, Fig. 6c), via some unknown, not-apoptosis related effect; in the absence of TrkA receptor, K252a failed to stimulate platelet production. These findings confirm that K252a modulates thrombopoiesis through inhibitions of TrkA signaling.

## Discussion

In this study, we investigated a novel regulatory role of TrkA signaling in megakaryopoiesis and thrombopoiesis. TrkA has previously been detected in CD34+ umbilical cord blood cells^28^ and our findings show that its expression is preserved throughout the MK lineage, in megakaryocytic cells lines as well as MKs differentiated from human primary CD34+ hematopoietic stem cells (henceforth termed as primary MKPs/MKs). In primary MKPs, treatment with NGF, a ligand for TrkA receptor, promoted expansion of the progenitor cells whereas exposure to K252a, a Trk inhibitor, reduced the numbers of both primary MKPs and MKs accompanied by a decrease in CD41 expression indicating that NGF-induced signaling drives the clonal expansion of the committed MKPs and is necessary for retainment of MK lineage markers^16^. Interestingly, NGF-mediated signals that are pro-mitogenic in progenitor cells, are not sufficient to induce MK expansion indicating that NGF asserts differential effects on mitotic MKPs versus endomitotic MK cells. Further pharmacologic inhibition of TrkA signaling via K252a decreased MKP proliferation and increased platelet production despite the presence of NGF. These findings demonstrate that TrkA signaling stimulates MKP proliferation and MK survival where as its inhibition induces platelet production by MK cells.

This role of TrkA signaling in megakaryocytic cells was further established via the use of CRISPR/CAS9 mediated knock out of TrkA in Dami cells. This cell line was found to recapitulate the effects of K252a on primary MKPs/MKs. Owing to the technical difficulty in conducting knock out experiments in primary cells, Dami cells were deemed to be a suitable substitute for performing these experiments. As expected, TrkA−/− Dami cells exhibited reduced proliferative capacity and increased production of platelet-like-particles, substantiating the function of TrkA signaling in this phenomenon. We also observed a positive correlation between the increase in platelet production and the percentage of apoptotic cells in TrkA-null Dami cells. Further, treatment of K252a to TrkA−/− cells, failed to increase PLP production, clearly indicating that K252a mediates its effects through inhibition of TrkA signaling. The link between localized activation of caspases and inhibition of proapoptotic proteins in platelet-forming MKs has been reported by multiple groups^63–67^. Besides, overexpression of anti-apoptotic proteins was shown to impair platelet production^68^.

In addition, we found that NGF induces activation of PI3K/Akt signaling pathway in a TrkA dependent manner in Dami cells. PI3K/Akt is known to mediate NGF-induced cell survival in neuronal cells and cardiomyocytes^69,70^. Upon NGF-mediated activation, Akt translocates to the nucleus and phosphorylates the proapoptotic transcription factor, Forkhead Box O3 (FOXO3)^70^. This leads to binding of 14-3-3 to FOXO3 and its sequestration in the cytoplasm^70^. Akt also promotes cell survival by inactivating Bad and preventing caspase 9 activation^70^. The same PI3K/Akt/FOXO3 signaling has been shown to mediate MK survival^64^. Therefore, Pi3K/Akt pathway might mediate decreased cell survival in K252a-treated or TrkA knockout cells which in turn drives increased platelet production.

Overall, we have shown that through activation of TrkA, NGF acts as a proliferation and/or pro-survival factor and supports early megakaryopoiesis but inhibits later platelet production from mature MKs. These findings might have several clinical implications for the treatment of hematologic disorders. Previous studies have reported that altered TrkA expression in myeloid and lymphoid leukemia provide either a proliferation or a survival advantage to CD34+ hematopoietic cells^71,72^. Therefore, TrkA was proposed as a therapeutic target for these malignancies^52,73^. In myeloproliferative disorders such as polycythemia vera or essential thrombocythemia, HSCs and early hematopoietic progenitors excessively proliferate due to increased cytokine sensitivity^74^, ultimately giving rise to increased MK and platelet production^75^. Besides altered TrkA signaling in myeloid malignancies, normal TrkA signaling can contribute to a myeloproliferative phenotype in the context of cytokine-hypersensitive HSCs and early hematopoietic progenitors. Hence, targeting TrkA, regardless of its normal or abnormal activity, might help to control excess proliferation of cells in myeloproliferative disorders.

In non-hematopoietic cancers, protection of MKPs against chemotherapy-induced cytotoxicity via the administration of hematopoietic cytokines (Stem Cell Factor [SCF], Interleukin 11 [IL-11] and thrombopoietin receptor agonists) has been previously proposed^76–78^. Given NGF’s role in MKP expansion and MK survival, NGF could be evaluated as a protective agent against chemotherapy-induced progenitor cell death in cancers with no TrkA involvement.

Lastly, patients receiving chemotherapy or undergoing myeloablative treatment for bone marrow transplantation experience thrombocytopenia due to the loss of hematopoietic stem and progenitor cells^76^. Recovery of complete blood cell counts takes approximately 20 days, which renders patients susceptible to infections and bleeding^79^. Platelet transfusion is the first line of treatment for severe thrombocytopenia in those patients^76,80,81^. However, limitations of platelet transfusion therapy include donor dependency, short platelet shelf-life, alloimmunization and infection risks^11,82–85^. The alternative approach to eliminate donor dependency and alloimmunization risk is the infusion of *ex vivo* differentiated MKPs or platelets generated from the patient’s own cells^86–88^. Current studies are investigating the best source of stem cells, timeline and order of cytokine addition to the culture in order to govern stem cell differentiation to MKs^88,89^. NGF/TrkA signaling can support these culture systems at an early stage where MKP enrichment is desired. Withdrawal of NGF or pulsing MKs with K252a analogs after MKs are generated might improve the platelet yield by promoting thrombopoiesis. Besides K252a, new TrkA antagonists, such as ALE0540, RN624, REN1820 are under development and are expected to provide TrkA-specific targeting^90–92^. Given the clinical potential of our findings, further in-depth investigation of the NGF-TrkA axis in thrombopoiesis using *in vivo* models is warranted. In addition, human platelets also possess TrkA expression on their cell surface (data not shown) and store NGF in their dense granules^93^. Hence investigations into the effects of NGF/TrkA signaling on platelet function might prove beneficial to prevent thrombotic events as well as to improve platelet storage by suppressing platelet activation.

## Supplementary information

Supplementary Dataset 1
