# Supplementary material for: Inhibition of Tropomyosin Receptor Kinase A Signaling Negatively Regulates Megakaryopoiesis and induces Thrombopoiesis
Source: Sci Rep. 2019 Feb 26;9:2781. doi: 10.1038/s41598-019-39385-x (PMC6391490; doi:10.1038/s41598-019-39385-x)
Supplement: Supplementary file 1 — Supplementary Dataset 1 [file 41598_2019_39385_MOESM1_ESM.pdf]

Inhibition of Tropomyosin Receptor Kinase A Signaling Negatively Regulates Megakaryopoiesis and Induces Thrombopoiesis

Ayse Kizilyer[1#,] Meera V. Singh[1], Vir B. Singh[1], Sumanun Suwunnakorn[1], James Palis[2], Sanjay B. Maggirwar[1*]

[1] Department of Microbiology and Immunology, University of Rochester Medical Center,

Rochester, NY, United States of America.

[2] Department of Pediatrics, Hematology and Oncology, University of Rochester Medical Center, Rochester, NY, United States of America.

[#] Current address: Department of Molecular Biology and Genetics, Faculty of Arts and Sciences, Burdur Mehmet Akif Ersoy University, Burdur, Turkey

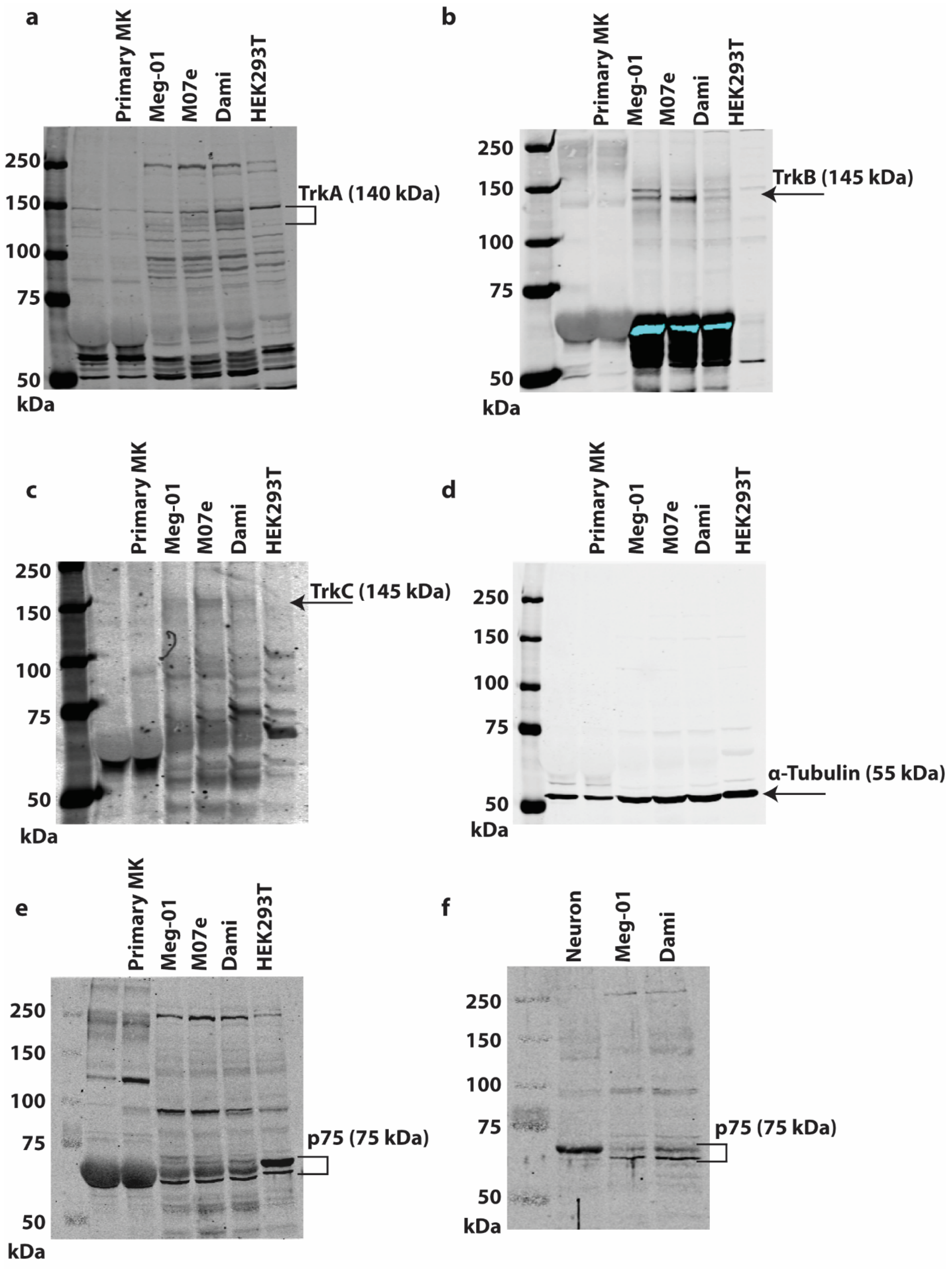

Supplementary Figure S1. Full-length blot images of Figure 1A. Neurotrophin receptors are differentially expressed both in human primary megakaryocytes (MKs) and human MK cell lines (Dami, Meg-01, and MO7e). Primary MKs were *ex vivo* differentiated from human umbilical cord blood-derived CD34+ cells. HEK293T cell and rat hypocampal neuron lysates were used as a positive control for neurotrophin receptor expression. For each receptor type, the same cell lysates were fractionated on a separate SDS-PAGE and processed in parallel. α-tubulin was used as a loading control. Molecular weight marker: Precision Plus Protein All Blue Prestained Protein Standards (Biorad)

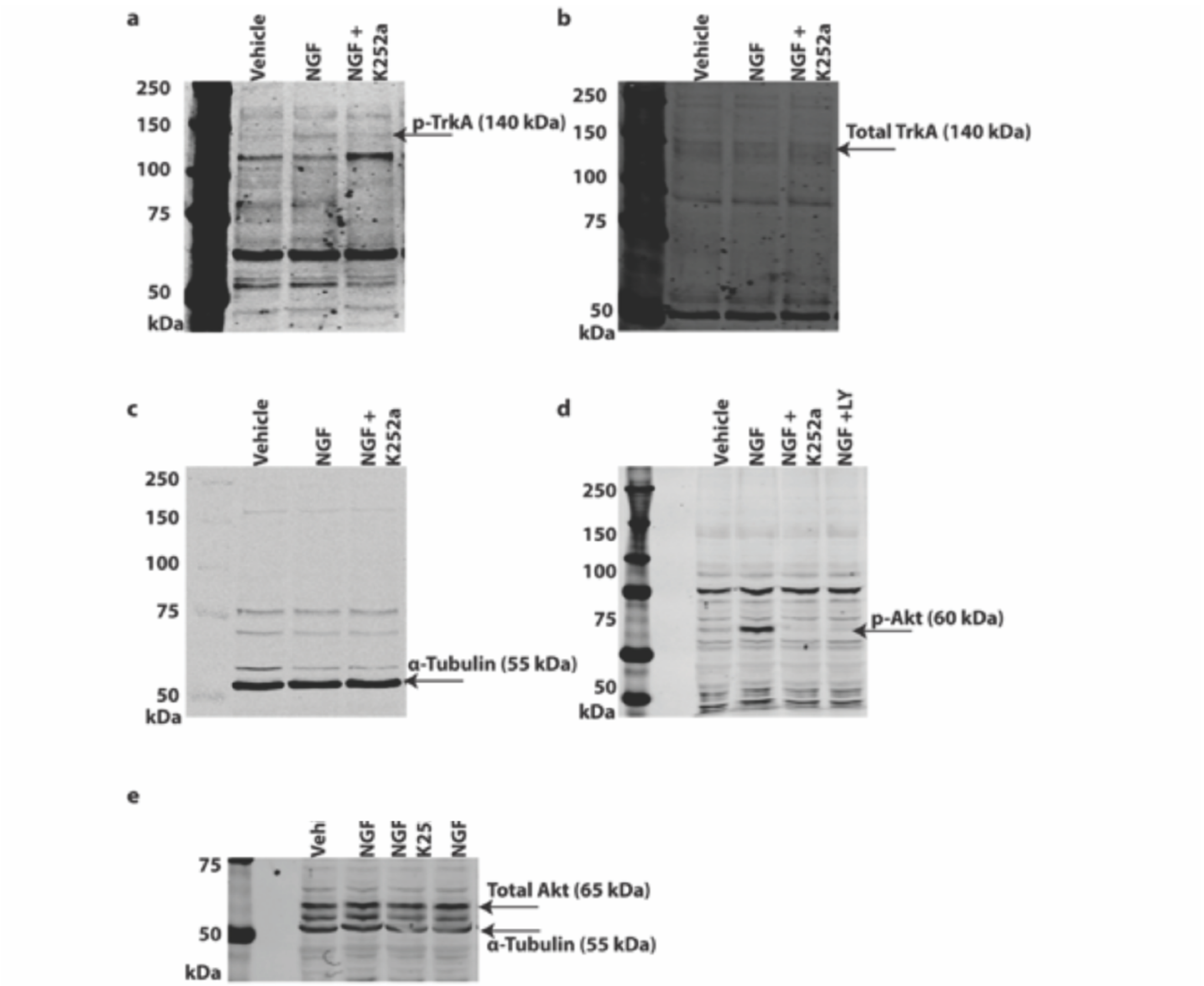

Supplementary Figure S2. Full-length blot images of Figure 3A and 3B. Dami cells serve as a model to study TrkA signaling. Dami cells were serum-starved for 1h and pre-treated with either 10 μM of LY294002 during serum starvation or 200 nM of K252a was added at the last 15 min of 1 h incubation. Then cells were incubated with 200 ng/ml rhNGF for 5 min. 30 ug of total cell lysates were analyzed by immunoblotting for **a)** phospho- and **b)** total TrkA. α-tubulin was used as a loading control **(c and e)**. The lysates were also analyzed by immnoblotting for **d)** phospho-Akt. The same blot was later stripped and reprobed for **e)** total Akt and α-tubulin, the loading control. Anti-rabbit and anti-mouse secondary antibodies labelled with separate infrared dyes were used to detect total Akt (red) and α-tubulin (green). NGF-induced TrkA signaling led to simultaneous phosphorylation of Akt whereas 15 min pre-treatment with 200 nM K252a blocked both TrkA and Akt phosphorylation. LY294002 pretreatment prevented phosphorylation of Akt, which indicates PI3K mediates NGF-induced Akt phosphorylation.

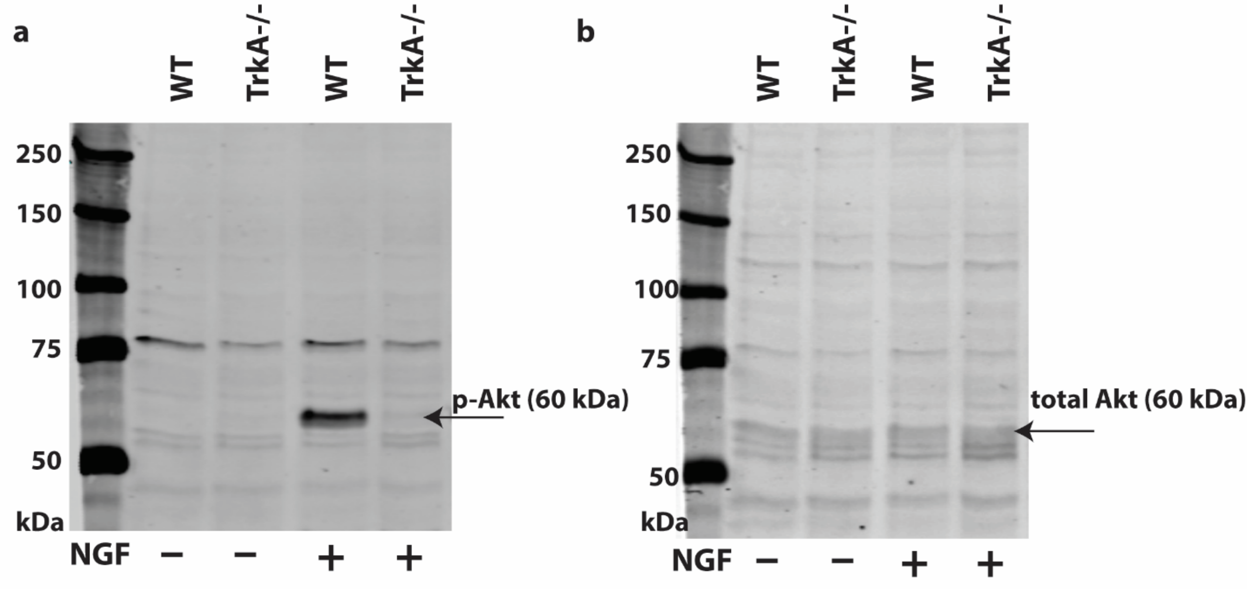

Supplementary Figure S3. Full-length blot images of Figure 5C. Functional validation of TrkA deletion by the loss of NGF-induced Akt phosphorylation in TrkA knockout (TrkA-/-) Dami cells. Both WT and TrkA-/- cells were subjected to serum starvation for 1 h and incubated with 200 nM NGF for 5 min. Cell lysates were prepared and analyzed by immunoblotting for **a)**phospho- and **b)** total Akt.

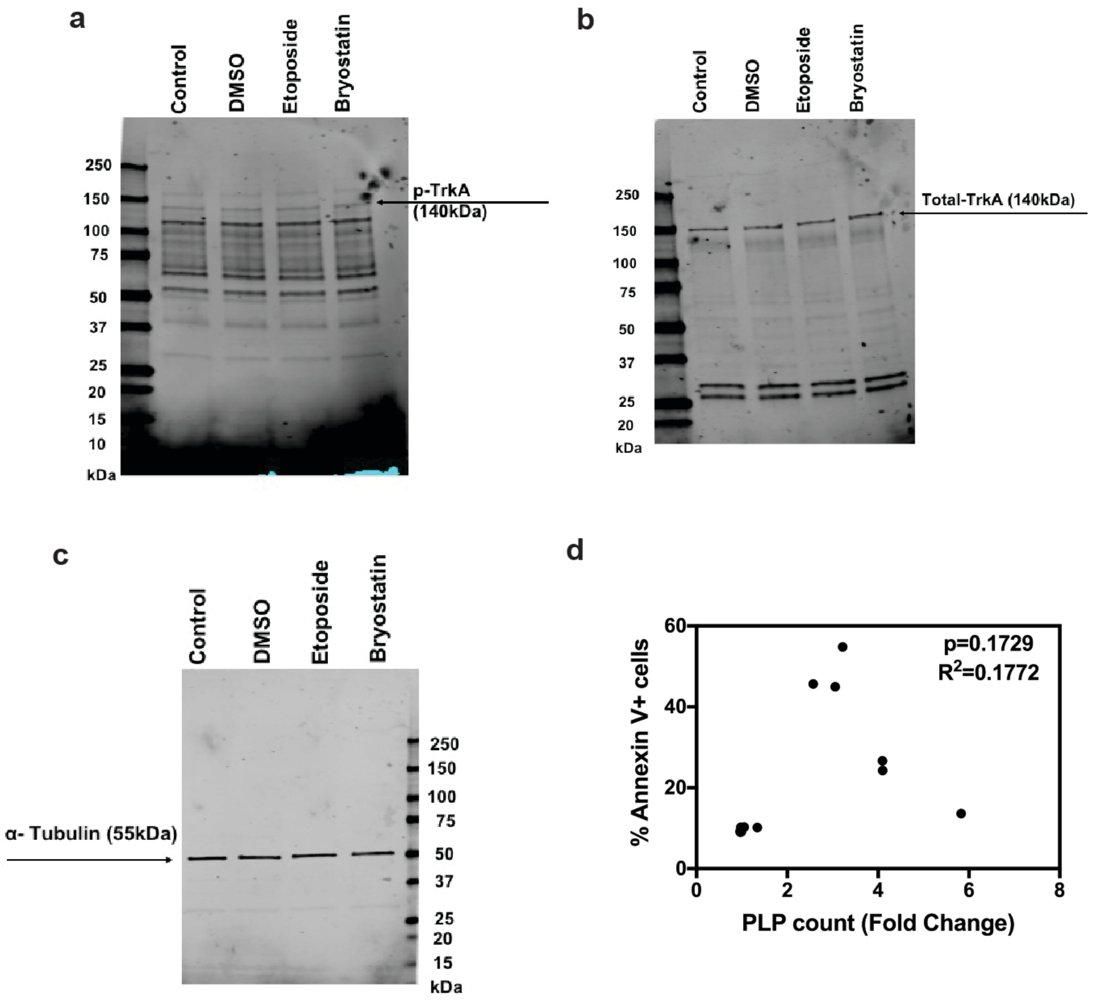

Supplementary Figure S4. a-c. Full-length blot images of Figure 4g for pTrkA, Total TrkA and Tubulin**. d.** Correlation data for percentage of Annexin V+ cells vs PLP counts (fold change).
